# Organosolv Lignin-Based Biopolyols Obtained via Oxyalkylation with Propylene Carbonate as Precursors of Rigid Polyurethane Foams

**DOI:** 10.3390/polym18131633

**Published:** 2026-06-30

**Authors:** Jacek Lubczak, Marzena Szpiłyk

**Affiliations:** Faculty of Chemistry, Rzeszów University of Technology, Al. Powstańców Warszawy 6, 35 959 Rzeszów, Poland; mszpilyk@prz.edu.pl

**Keywords:** organosolv lignin, glycidol, glycerol, propylene carbonate, polyols, polyurethane foams, properties

## Abstract

The study presents the results of research on the preparation of biopolyols based on organosolv lignin and their application in the synthesis of rigid polyurethane foams. The research was conducted in order to develop a sustainable alternative to the previously used ethylene carbonate in lignin oxyalkylation processes. The main objective was to replace the previously used ethylene carbonate with propylene carbonate in a stoichiometrically equivalent molar amount in order to reduce polyol viscosity and improve the performance properties of the resulting foams. The syntheses were carried out without the need for isolation and purification of intermediate products. Polyols analogous to those described previously were obtained and subsequently used for the preparation of rigid polyurethane foams employing polymeric diphenylmethane diisocyanate. The properties of the obtained foams were investigated and compared with those of foams prepared from ethylene carbonate-based polyols. The results demonstrated that the use of propylene carbonate leads to the formation of lower-viscosity polyols, facilitating homogenization of the reaction systems and enabling the production of foams with advantageous performance characteristics, generally superior to those of foams based on ethylene carbonate. The obtained materials constitute a promising alternative to conventional polyurethane foams derived from petrochemical raw materials.

## 1. Introduction

Replacing petroleum-derived polyols with products derived from natural resources is currently a significant development direction for the modern chemical industry, aligning with the concept of sustainable development. This approach reduces the consumption of non-renewable resources, mitigates the negative environmental impact, and reduces greenhouse gas emissions. Furthermore, the use of natural resources, such as vegetable oils or biomass by-products, supports the development of the bioeconomy and can lead to materials with comparable or even superior performance compared with their petrochemical counterparts [1].

Lignin is the second most common biopolymer in nature after cellulose and is one of the main components of the cell walls of woody plants [2]. Due to its aromatic structure and high hydroxyl group content, it is a promising raw material for obtaining biopolyols, which can be used as an alternative to petrochemical polyols. There are relatively few reports in the literature on attempts to obtain polyols and from them polyurethane foams containing lignin [3,4,5]. This is primarily due to its insolubility in organic solvents, in which it would be possible to conduct reactions with oxiranes or alkylene carbonates, aimed at obtaining polyols suitable for the production of polyurethane foams. Reported reactions of lignin with oxiranes led to the formation of powdery or semi-solid resins, unsuitable for further conversion into polyurethane foams due to their lack of miscibility with liquid isocyanates [6,7,8,9,10]. Furthermore, attempts at the oxyalkylation of lignin with alkylene carbonates required maleic anhydride in order to improve lignin solubility [2,11]. Several studies have reported the preparation of polyols by dispersing lignin in organic liquids such as polyethylene glycol and glycerol [12], castor oil [13], and propylene carbonate, which acts as a green co-solvent [14]. In another procedure, the alkylene carbonates (ethylene, propylene, and butylene carbonates) were used as oxyalkylating agents for lignin in poly(ethylene glycol) or glycerol in the presence of sulfuric(VI) acid [15,16]. Previous studies have reported the synthesis of lignin-derived polyols through the reaction of lignin with a tenfold molar excess of a cyclic carbonate. The reaction conditions, including catalyst type, temperature, and reaction duration, were optimized to maximize product formation. Following synthesis, the products were isolated by precipitation in acidified water, collected by filtration, and dried under reduced pressure in the presence of P_2_O_5_. However, the resulting polyols were obtained as solids, which complicated subsequent purification steps.

In one approach, lignin was liquefied by reaction with poly(ethylene glycol) and glycerol at 120 °C using sulfuric acid as a catalyst. Purification involved removing insoluble residues from a dioxane–water solution, followed by solvent evaporation. Polyurethane foams prepared from these lignin-containing polyols exhibited enhanced resistance to both thermal degradation and thermo-oxidative aging compared with foams produced without lignin-derived components.

An alternative liquefaction route was reported in which lignin and glycerol were combined at a mass ratio of 1:9 and reacted at 160 °C for 90 min in the presence of sulfuric acid [16]. The reaction mixture was subsequently diluted with ethanol and stirred for an additional 5 h. After filtration to remove insoluble fractions, the solvent was evaporated to obtain liquid lignin-based polyols suitable for further polyurethane synthesis.

Several studies have explored the partial substitution of petrochemical-based polyols with either untreated lignin powder [17] or sulfomethylated lignin [18]. Although these approaches enable the production of flexible polyurethane foams, they are associated with certain limitations. In the case of untreated lignin powder, the addition of NaCl is necessary to control and optimize the cellular structure of the foam. Conversely, the use of sulfomethylated lignin requires a multistep modification process involving treatment with formaldehyde and sodium sulfite (Na_2_SO_3_), followed by purification. The primary objective of this modification is to improve the solubility of lignin in PEG200.

The main obstacle to obtaining the final polyols from lignin was the multistep procedure and the complex methods required for the separation and purification of the products. Despite numerous studies on lignin-based polyurethane materials, several challenges remain unresolved. These include the limited solubility of lignin, the high viscosity of lignin-derived polyols, the need for multistep synthesis and purification procedures, and the difficulty of obtaining polyurethane foams with simultaneously low density, low water uptake, and high thermal resistance. However, we have recently obtained polyols from lignin organosolv (OL) by oxyalkylation while avoiding the tedious removal of solvents and solid residues and subsequently used the polyols to obtain rigid polyurethane foams [19,20].

First, we heated the mixture of OL, glycidol (GL), and ethylene carbonate (EC) at 170 °C until the reaction between OL and GL was completed. Then, potassium carbonate was added as a catalyst, and the reaction with EC was continued at 160–170 °C [19]. In another procedure, the reaction of OL with EC was conducted in glycerol (GLYC) in the presence of potassium carbonate at 150–160 °C [20]. In both methods, liquid polyols were obtained and then used to produce rigid polyurethane foams.

Three variants of polyol syntheses with OL, GL, and EC were tested. In the synthesis of polyol A, the minimal amount of GL and EC required for the dissolution of OL was used. The product obtained in this case was a very viscous polyol. In order to obtain a less viscous polyol, the amounts of GL and EC were increased, and polyols B and C were obtained. Similarly, the reaction of OL with EC was performed in GLYC to obtain polyols X, Y, and Z [20].

The proportions of the individual components were selected in such a way that the viscosity of the polyol allowed good mixing with polymeric methylene diphenyl diisocyanate (pMDI), while at the same time ensuring that the resulting foam did not exhibit brittleness, which could occur if the OL content in the polyol was too high.

The weight ratios of the reagents used in the corresponding procedures were as follows (OL/GL/EC): A—1.00/3.75/10.00; B—1.00/3.75/12.50; C—1.00/5.00/10.00. In the second method the ratios were as follows (OL/GLYC/EC): X—1.00/2.00/8.00; Y—1.00/2.00/12.00; Z—1.00/4.00/8.00. The PUFs were obtained from the synthesized polyols. We found that the obtained PUFs were similar to traditional rigid PUFs and, additionally, exhibited good or very good thermal resistance. Additionally, they appeared to be easily biodegradable in soil.

Continuing the previously initiated research on the use of lignin for the preparation of polyols and polyurethane foams, we decided to carry out reactions involving propylene carbonate (PC). Earlier studies [21] had shown that replacing EC with PC, while maintaining the same molar ratio, led to the formation of polyols with lower viscosity. This facilitated the homogenization of the foaming compositions and allowed the production of foams with more favorable performance properties, including lower apparent density and higher mechanical strength.

Analyzing the most favorable functional properties of the polyurethane foams described in the publications [19,20], it was proposed to obtain equivalents of polyols B, C, Y, and Z by replacing EC with a stoichiometrically equivalent molar amount of PC. The obtained PC-derived polyols, named B′, C′, Y′, and Z′, were then foamed with diphenylmethane diisocyanate (pMDI) to obtain PUFs. The physical properties of the PUFs were studied in detail and compared with those of the EC-derived analogues, as described previously [19,20].

The novelty of the present work lies in the use of propylene carbonate as a direct substitute for ethylene carbonate in previously developed solvent-free oxyalkylation routes of organosolv lignin. This approach enables the preparation of lignin-based polyols with lower viscosity without the isolation of intermediates or purification steps. In addition, the work provides a systematic comparison between polyurethane foams obtained from EC- and PC-derived polyols, allowing the influence of polyol structure on foam performance to be evaluated.

## 2. Materials and Method

### 2.1. Materials

The following materials were used in this work: OL (90%, BOC Sciences, Shirley, NY, USA); GL (pure 98%; Sigma-Aldrich, Taufkirchen, Germany); GLYC (pure 97.5%; Avantor Performance Materials, Gliwice, Poland), PC (pure, ≥99%; Fluka, Buchs, Switzerland); potassium carbonate (anal. grade 100%; POCH, Gliwice, Poland); polymeric diphenylmethane 4,4′-diisocyanate (pMDI; Merck, Darmstadt, Germany); triethylamine (TEA, anal. grade ≥ 99%; Fluka, Buchs, Switzerland); and surfactant Silicon L-6900 (pure; Momentive, Wilton, CT, USA).

### 2.2. Synthesis of Polyols

#### 2.2.1. Synthesis with GL 24 g of OL

A total of 90 g of GL and 347.7 g of PC were placed in a 500 cm^3^ three-necked round bottom flask equipped with a reflux condenser, mechanical stirrer, and thermometer. The mixture was stirred at a speed of 1000 rpm, and the temperature was raised gradually to 175–185 °C until GL was completely consumed, based on the determination of the epoxide number. Then the mixture was cooled down to 70 °C, and 1.5 g of potassium carbonate was added as a catalyst. The mixture was again heated to 170 °C with vigorous stirring until PC had completely reacted, as determined by monitoring the PC concentration in the product. During the reaction, intense release of CO_2_ occurred due to the decomposition of PC. The dark brown resin (polyol B’) was obtained. Under analogous conditions, another synthesis was performed using 24 g of OL, 120 g of GL, and 278.2 g of PC (polyol C′). The products were obtained with the yields of: B′—273.8 g (87.3%) and C′—262.2 g (84.7%).

#### 2.2.2. Synthesis with GLYC

A total of 20 g of OL, 40 g of GLYC, and 278.2 g of PC were placed in a 500 cm^3^ three-necked round bottom flask equipped with a reflux condenser, mechanical stirrer, and thermometer. The mixture was stirred at a speed of 1000 rpm and heated gradually to 170 °C until OL was dissolved. Then, 1.5 g of potassium carbonate was added, and the mixture was heated at 175–180 °C while being stirred until PC was completely consumed, as determined by its concentration in the product. A dark brown resin (polyol Y′) was obtained. Another synthesis was performed in an analogous way starting with 20 g of OL, 80 g of GLYC, and 185.5 g of PC (polyol Z″). The yields were: Y′—178.2 g (81.1%) and Z′—163.5 g (79.0%).

### 2.3. Analytical Methods

The determination of aliphatic and phenolic hydroxy groups, as well as methoxy groups and weight-average molecular weight (Mw) of OL was performed as described in detail in [19,20]. The progress of the reaction between OL and GL was followed by determination of the epoxide number [22]. The hydroxyl number of the polyol was determined by acylation with acetic anhydride in dimethylformamide [23]. Oxyalkylation with PC was monitored using the barium hydroxide method described in [24]. The ^1^H-NMR spectra of the reagents were recorded at 500 MHz using a Bruker UltraShield instrument (Bruker, Billerica, MA, USA) in DMSO-d_6_ and D_2_O with hexamethyldisiloxane as an internal standard. IR spectra were registered on an ALPHA FT-IR BRUKER spectrometer (Bruker, Billerica, MA, USA) in KBr pellets or by the ATR technique. MALDI–ToF spectra of the polyols were obtained on a Voyager-Elite Perceptive Biosystems (US) mass spectrometer working in linear mode with delayed ion extraction, equipped with a nitrogen laser working at 352 nm. The method of laser desorption from gold nanoparticles (AuNPET LDI MS) was applied [25]. Some observed peaks corresponded to the molecular ions K^+^ (from catalyst). The weight-average molecular weight (Mw) of OL was determined by gel permeation chromatography (GPC) using a simplified Shimadzu (Kyoto, Japan) system consisting of an LC-20AT isocratic pump, a manual Rheodyne injector, and a RID-10A refractive index detector. The separation was carried out using a single PSS GRAM column (3000 Å, stainless steel) suitable for the expected molecular weight range. The eluent was N,N-dimethylformamide (HPLC grade) containing 0.01 M LiCl, with a constant flow rate of 1 mL/min. The column temperature was maintained at room temperature. Calibration was performed using commercial monodisperse polystyrene standards (PSS Polymer Standards Service, Mainz, Germany).

### 2.4. Physical Properties of Polyol

Density, viscosity, and surface tension of polyols were determined with a pycnometer, a Höppler viscometer (typ BHZ, prod. Prüfgeratewerk, Germany) and by the detaching ring method, respectively.

### 2.5. Obtaining the Polyurethane Foams

Foaming of polyols was performed in 500 cm^3^ cups at room temperature. The foams were prepared from 10 g of polyol, to which 0.130–0.155 g of surfactant (Silicon L-6900) and 0.035–0.090 g of TEA as a catalyst, and water (2–3%) as a blowing agent were added. After homogenization, pMDI was added in the amount of 20.0–26.3 g. A commercial isocyanate containing 30% of tri-functional isocyanates was used. The mixture was vigorously stirred until creaming began. The materials were then seasoned at room temperature for 3 days. The samples for further studies were cut from the obtained foam.

### 2.6. Properties of Foams

The apparent density [26], water absorption [27], dimensional stability at a temperature of 150 °C [28], heat conductance coefficient (IZOMET 2104, Applied Precision, Rača, Slovakia), and compressive strength [29] of the PUF were measured. The methods used are described in detail in previous works [19,20]. The heat conductance coefficient was measured at 20 °C after 72 h of PUF conditioning. The needle was inserted 8 cm deep into a cylindrical PUF sample with dimensions 8 cm in diameter and 9 cm in length during the measurement. Compressive strength was determined using a load corresponding to 10% compression of the PUF height relative to its initial height (in accordance with the PUF growing direction). The thermal resistance of the foams was determined using both static and dynamic methods. In the static method, the foams were heated at 150 and 175 °C with continuous measurement of mass loss and determination of mechanical properties before and after heat exposure. The cubic samples with dimensions of 100 × 100 × 100 mm were used to determine the static thermal resistance and compressive strength. In the dynamic method, thermal analyses of the foams were performed in a ceramic crucible over the temperature range of 20–600 °C, with about 100 mg of sample, under an air atmosphere using a TGA/DSC 1 thermobalance derivatograph (Mettler Toledo, Greifensee, Switzerland), Mettler, with a heating and cooling rate of 10 °C/min. Topological images of the PUFs were recorded for cross-sections of the PUFs samples. The microstructure of the pore structures was analyzed by scanning electron microscopy using a ZEISS GeminiSEM microscope. A Quorum Technologies Q150T E Plus (Quorum Technologies, Lewes, UK) high-vacuum sputter coater was used to coat the samples. A 120 s sputtering program was used to create a chromium nanolayer (0.2 nm) on the sample surface. The studies were performed at an accelerating voltage ranging from 5 to 10 kV, with magnifications of 100×. Statistical analysis of the pores was carried out on the basis of the obtained micrographs using ImageJ software (SmartSEM version 8.00).

## 3. Results and Discussion

### 3.1. Synthesis and Properties of Polyols

The lignin substrate OL was previously characterized and successfully used to obtain polyols by reactions with EC [19,20]. In this case, EC was replaced with PC in an equivalent molar ratio, using formulations analogous to those for EC. The main difficulty in preparing liquid polyols from lignin is its limited solubility. We found that OL is insoluble in PC. Moreover, heating OL in PC at 150–180 °C in the presence of K_2_CO_3_ leads to gelation of the mixture. Our previous studies showed that GL is a very convenient reactive solvent for polymers containing reactive hydroxyl groups. We attempted to dissolve lignin in GL and obtained a semi-solid resin that was readily soluble in hot water. We also observed that after the reaction of lignin with GL, no unreacted solid lignin remained. However, the high viscosity of the resulting resin prevented proper miscibility with pMDI and hindered the formation of polyurethane foams (PUFs).

The simultaneous use of these oxyalkylating agents is beneficial, as intermediate products formed during the reaction with GL gradually become soluble in PC. The reaction with GL proceeds without a catalyst. The consumption of GL was monitored by measuring the epoxy value. After complete consumption of GL, K_2_CO_3_ was added to catalyze the subsequent reaction with PC. Determination of the propylene carbonate content after the reaction of lignin with GL showed that, in the absence of potassium carbonate, PC is essentially non-reactive and acts only as a reaction medium (only 0.9% of PC relative to its initial amount was consumed during the reaction of OL with GL).

Two pairs of syntheses were carried out using OL, GL, and PC, as well as OL, GLYC, and PC.

**Scheme 1:**
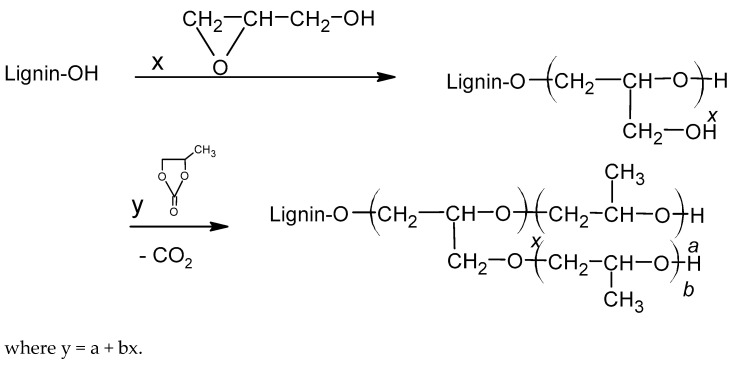
General scheme for obtaining polyol by reactions of OL with GL and PC shown on a structural fragment of lignin.

**Figure 1:**
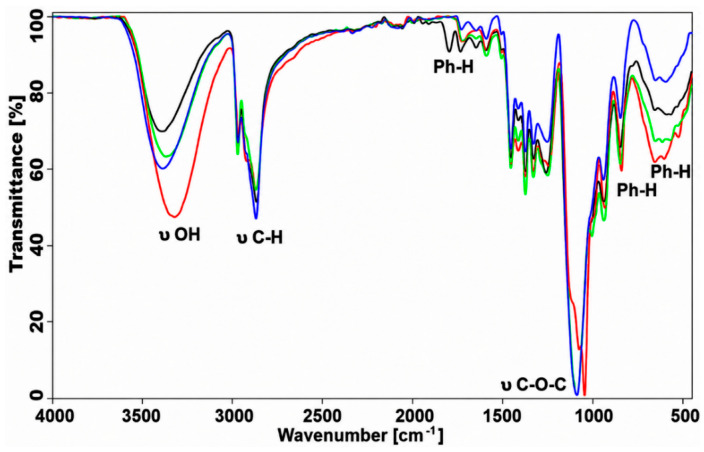
IR spectrum of polyols B′ (black line), C′ (blue line), Y′ (green line) and Z′ (red line).

In the syntheses of polyols B′ and C′, different weight proportions of GL and PC were applied, and in the syntheses of polyols Y′ and Z′, different proportions of GLYC and PC were used. Generally, the reaction time was within 5 h, and the temperature was within the range of 175–185 °C. The obtained polyols were dark brown resins. Their density, viscosity, and surface tensions were within the ranges typical of polyols used to obtain PUFs [30] (Table 1). It was found that polyols obtained from OL, GL, and PC had higher viscosity than those polyols obtained from OL, GLYC, and PC. This was presumably due to the higher number of intermolecular hydrogen bonds, because each step of the reaction with GL increased the functionality of the product by 1. This is also indicated by the broadening of the band corresponding to the stretching vibrations of hydroxyl groups at 3300 cm^−1^ in the IR spectra of the obtained polyols (Figure 1). The determined molecular weights (Mw) of the polyols ranged from 21,247 to 76,370 u. Polyol C′, synthesized with the highest GL content, exhibited the highest molecular weight and, consequently, the highest viscosity. In contrast, polyols Y′ and Z′ showed lower Mw values, which can be attributed to the reaction being conducted in a GLYC medium. The hydroxyl numbers of the polyols were ca. 400 mg KOH/g or higher, indicating their suitability for the preparation of rigid PUFs. The small values of the acidic number of the obtained polyols indicate the possibility of incomplete oxyalkylation of the phenolic groups, as demonstrated in the work [20]. During the reaction of OL with PC, mainly secondary hydroxyl groups are formed (Scheme 1), whereas syntheses involving EC form primary hydroxyl groups [19,20].

**Scheme 2:**
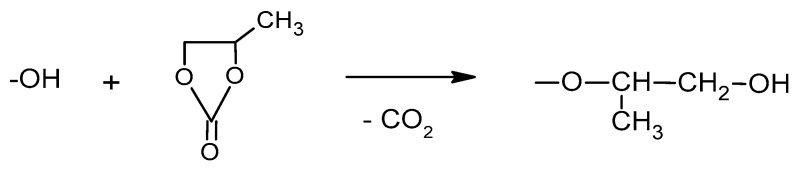
PC reaction with the formation of primary hydroxyl groups.

However, the formation of primary hydroxyl groups cannot be excluded, which is related to the different ring opening pathways of PC (Scheme 2).

**Scheme 3:**
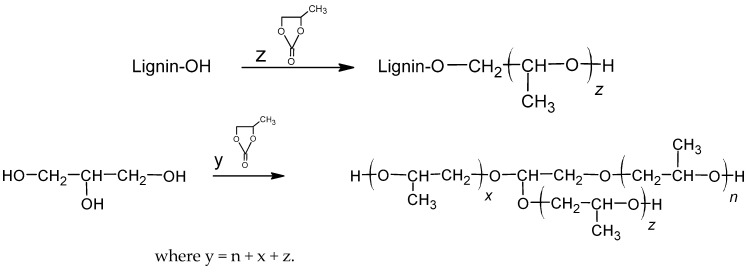
Obtaining of polyols from OL and PC in GLYC medium.

In the method with GL and PC, the reaction was initially performed between OL and GL in PC as a solvent, while after the reaction was completed, potassium carbonate was added and the oxyalkylation with PC was continued. In the second method, after dissolving OL in GLYC, the reaction with PC was carried out directly. Scheme 3 illustrates the reactions of PC using a lignin structural fragment as an example. The parallel reactions involving GLYC are shown below. During all syntheses, CO_2_ evolution was observed.

**Scheme 4:**
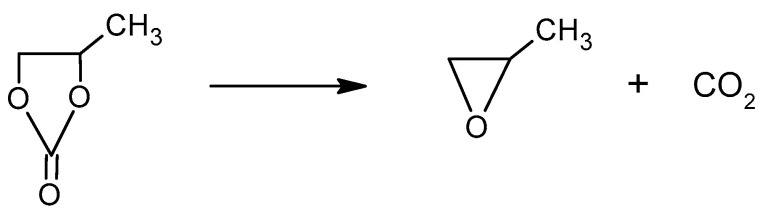
Thermal decomposition of PC.

The synthesis was stopped after the gas evolution had ceased and the propylene carbonate content in the reaction mixture was found to be below 0.5 wt%. Weight analysis (substrates vs. products) indicated that mass loss during the reaction was higher than that calculated for the complete elimination of CO_2_ from PC. This means that the number of oxypropyl groups in the product was lower than the number of PC equivalents. This suggested that PC partially decomposed into volatile CO_2_ and propylene oxide in the reaction conditions (Scheme 4) [21,30].

**Scheme 5:**
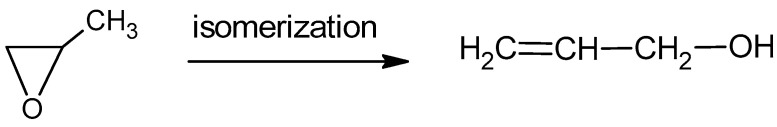
Isomerization of PC.

Moreover, newly formed propylene oxide could isomerize into allyl alcohol [21] (AA), which contributed to the product’s characteristic odor (Scheme 5).

**Scheme 6:**
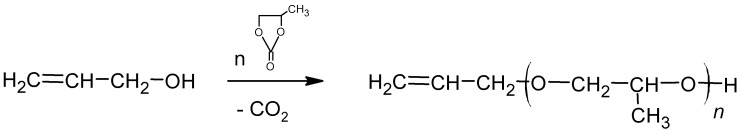
Oxyalkylation of AA by PC.

In the conditions of the synthesis, AA undergoes oxyalkylation with PC, therefore its odor gradually disappears (Scheme 6).

In order to detect AA in the product, we attempted to isolate it by distillation. We quantified the amount of AA as 2% relative to the mass of the polyol. AA was identified by IR and ^1^H-NMR spectroscopies, as well as by determination of the refractive index.

The products were analyzed using IR, ^1^H-NMR and MALDI–ToF techniques. The IR and H-NMR spectra of OL have been described in detail previously [19,20]. In the spectra of polyols obtained from OL (Figure 1), strong bands were observed in the range of 1050–1060 cm^−1^, related to C-O-C bonds formed upon reaction of the hydroxyl groups of OL with GL and PC.

**Scheme 7:**
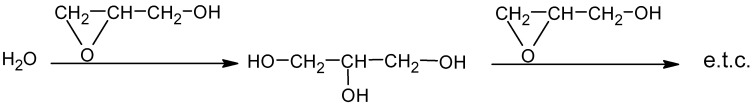
Formation of GLYC and consecutive products during the reaction of water with GL.

**Scheme 8:**
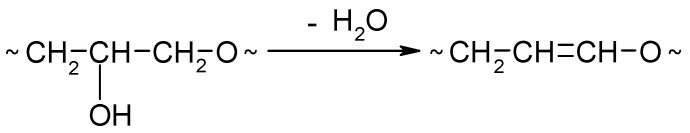
Dehydration of oligomers and polyols.

**Figure 2:**
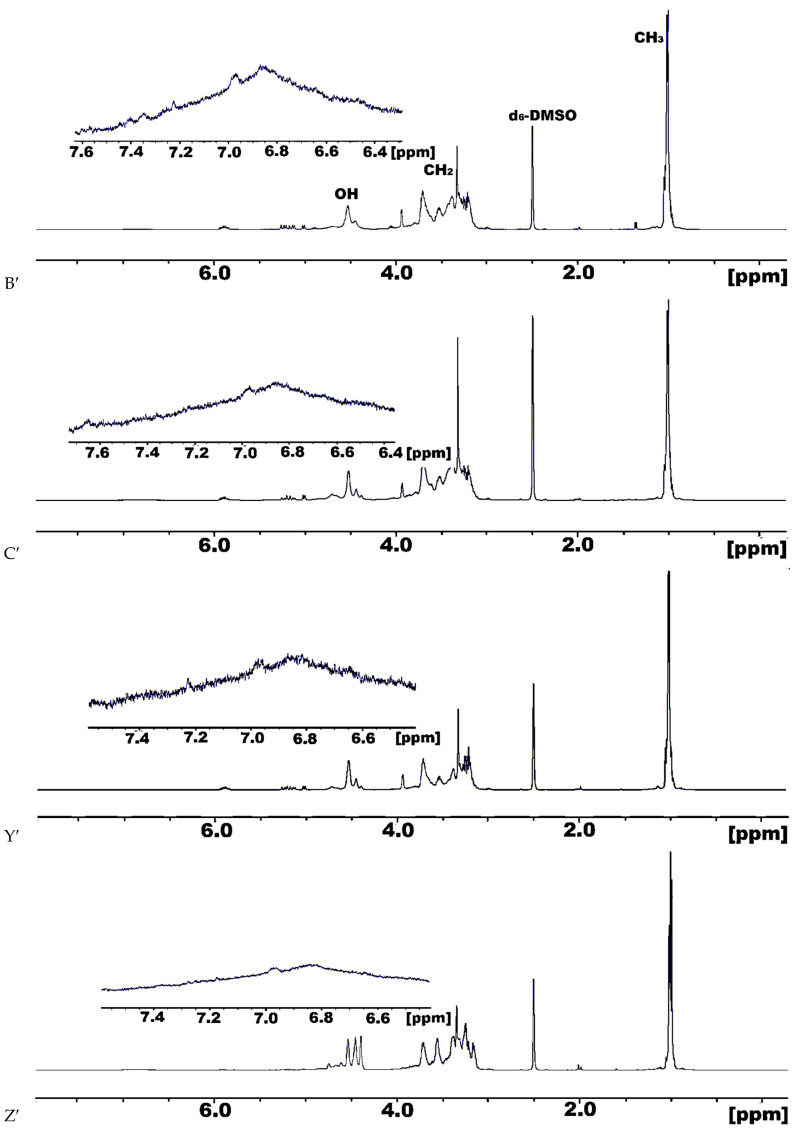
H-NMR spectrum of polyols B′, C′, Y′ and Z′.

This provides evidence for the oxyalkylation of lignin and the incorporation of fragments from the ring opening of GL and PC into the polyol structure. The complete absence of the ester band at 1250 cm^−1^ indicated that PC reacted with concomitant elimination of CO_2_ without the incorporation of ester groups into the polyol. Furthermore, an increase in the intensity of the band centered at 2870 cm^−1^ was observed and attributed to newly formed methylene, methine, and methyl groups resulting from PC ring opening during synthesis. The ^1^H-NMR spectrum of OL was presented and analyzed previously [19,20]. The ^1^H-NMR spectra of the obtained polyols are presented at Figure 2. The carboxyl proton resonance of OL was previously observed at 12 ppm, which was absent in the spectra of the obtained polyols, indicating the involvement of the carboxyl group in the reaction with GL and/or PC. The hydroxyl proton resonance of OL overlaps with methylene and methine proton resonances within the range of 3.5–4.2 ppm. The intensity of this resonance dropped significantly in the spectra of the polyols due to the reaction of the OH groups of lignin with GL and/or PC. Instead, new hydroxyl resonances were formed, giving rise to a signal at 4.5 ppm (confirmed by selectively deuteration with D_2_O). Newly formed methylene and methine groups from GL and PC ring opening and addition to OL give a series of resonances at 3.1–3.5 ppm. Furthermore, PC ring opening is accompanied by a signal at 1.0 ppm due to formation of methyl groups, according to Scheme 1. A low intensity resonance at 1.1 ppm also originates from a methyl group formed via an alternative PC ring-opening pathway, as shown in Scheme 2. The structure of lignin remains unchanged, as shown by the resonances within the range 6.4–7.6 ppm (see the insert in Figure 2), which are also present in the spectrum of OL. Low-intensity resonances within the range of 4.6–6.0 ppm can be observed in the spectra of the polyols. These can be attributed to olefinic protons, which are consistent with the formation of AA and products of its oxyalkylation, as mentioned above. The MALDI–ToF spectra of the obtained polyols also show peaks corresponding to AA (Table 2 and Table 3, entries 1 and 2), as well as peaks corresponding to the products of its oxyalkylation with PC (Scheme 6, Table 2, entries 6, 13, 14, and 18; Table 3, entries 6, 7, 13, 15, 16, 25). The spectra of polyols obtained from OL, GL, and PC show the peaks of products formed by reaction of water with GL, leading upon first reaction into GLYC (Scheme 7, Table 2 entry 3), and then to consecutive products formed upon further reaction with GL (Table 2, entries 8, 16). Water originates both from the lignin and from its formation in the reaction mixture through the dehydration of oligomers and polyols (Scheme 8, Table 2, entries 9, 10, 17, 23, 26, 32 and Table 3, entries 10, 18, 21, 24) at the high temperature at which oxyalkylation of lignin is performed.

GLYC can then react with PC to form the appropriate oligomers (Scheme 3, Table 2, entries 5, 11, 19, 20, 25, 27, 29, 31).

**Scheme 9:**
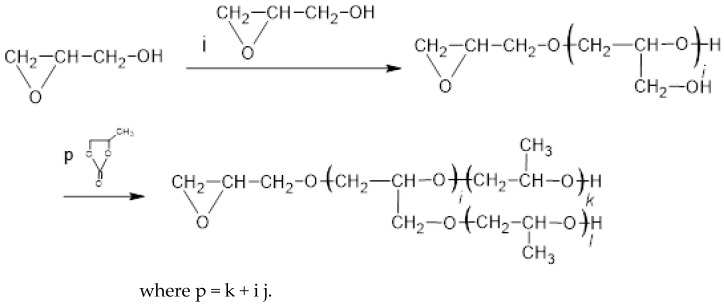
Oligomerization of GL and reactions with PC.

In the case of reactions conducted with a mixture of GL and PC, the oligomers of GL can be formed, which consecutively react with PC (Scheme 9, Table 2, entries 4, 7, 12, 15, 21, 22, 24, 28, 30, 33):

In the reactions performed in GLYC, low-molecular-weight products of PC oxyalkylation were observed (Scheme 3, Table 3, entries 5, 9, 11, 14, 17, 19, 20, 22, 23, 26).

### 3.2. Polyurethane Foams

The obtained polyols were used to fabricate rigid polyurethane foams (PUFs). The PUFs were obtained by the reaction of the polyol with pMDI, with TEA as a catalyst and Silicon L-6900 as a surfactant. The carbon dioxide released from the condensed phase played the role of a blowing agent and was formed during the reaction of water with pMDI. The optimized water amount was 2 or 3 wt% relative to the mass of the polyol. Larger amounts of water resulted in the formation of PUFs with irregular pores and low compressive strength, while less than 2% water formed PUFs with high apparent density.

The foaming process was optimized (Table 4). The criterion was the rigidity and regular cellular structure of the PUF. Based on the determined hydroxyl number, the isocyanate coefficient defined as the molar ratio of isocyanate groups to hydroxyl groups (–NCO/–OH) in the initial reaction mixture, was calculated. Subsequently, this value was optimized experimentally and found to be larger than the calculated value, namely 1.8–2.1. This suggested that partial trimerization of isocyanates took place in the reaction mixture, leading to the formation of perhydro-1,3,5-triazine rings. This was confirmed by the detected deformation band of the NH group and the C=O band in the IR spectrum of the obtained foams, at 510 and 770 cm^−1^, similarly to that reported in [20], as well as by their high thermal resistance. When the amount of pMDI, in relation to the experimentally determined isocyanate coefficient, was decreased, it resulted in the formation of PUFs, that were dimensionally unstable after heating to 40 °C or even after a couple of days of storage at room temperature. Finally, we found that the optimal amount of the surfactant Silicon L-6900 was 1.00–1.55 g, while the amount of TEA catalyst should be kept within the range of 0.35 to 0.90 g per 100 g of polyol in the foaming composition (Table 4). A higher amount of catalyst than that mentioned resulted in a shorter rise time and disruption of the formed PUF, while a lower amount of TEA resulted in under-crosslinked semi-rigid PUFs with a sticky surface.

Cream time was within the range of 40–65 s except for composition C’ and Z’ containing, respectively, 2 or 3 g water per 100 g of polyol, for which the creaming times were 90 s. The rise time ranged from about one to two minutes and the drying time was within a few seconds.

The following properties of obtained PUFs were determined: apparent density, water uptake by volume, dimensional stability, heat conductance coefficient, heat resistance, compressive strength and glass transition temperature. Apparent density of PUFs ranged from 47.8 to 53.6 kg/m^3^ and from 39.1 to 54.1 kg/m^3^ for PUFs obtained with 2 or 3 g water per 100 g polyol, respectively (Table 5). The PUFs obtained with GL contribution exhibited higher apparent density. This is due to higher probability of formation of branched chains in the obtained polyol, resulting in more rigid and better crosslinked PUF. Furthermore, the high viscosity of the polyol limits the gas diffusion and hampers the stable growth of bubbles and ultimately leads to reduced foaming.

Synthesis of polyols in GLYC reduces the aforementioned obstacles; the viscosity of the polyol is lower, the PUF is less rigid due to formation of longer oxypropyl chains attached to GLYC, which finally leads to PUFs of lower apparent density. Water uptake by volume of PUFs obtained from Y′ and Z′ polyols ranges from 0.53 to 0.60% after 24 h exposure to water (Table 5). This may be important for applications in the building industry, in which the water uptake of such materials should not exceed 3–5%. Low values of water uptake of the obtained PUFs suggest a closed-cell pore structure. The PUFs obtained from polyol with GL contribution have higher water uptake, namely 2.2–4.9%. This is probably due to the presence of many hydrophilic centers, which are formed upon GL ring-opening and its reaction with isocyanate.

These PUFs show good dimensional stability at elevated temperatures (Table 5). Their maximal shrinkage after 40 h of thermal exposure at 150 °C is within 0.20 to 2.65%. The exception is the PUFs obtained from C′ polyol with 3 g water per 100 g polyol in the foaming composition for which the shrinkage reached 4.8%. Thermal conductivity coefficient was typical for rigid PUF and fell within the 0.0236–0.0267 W/m·K range.

**Figure 3:**
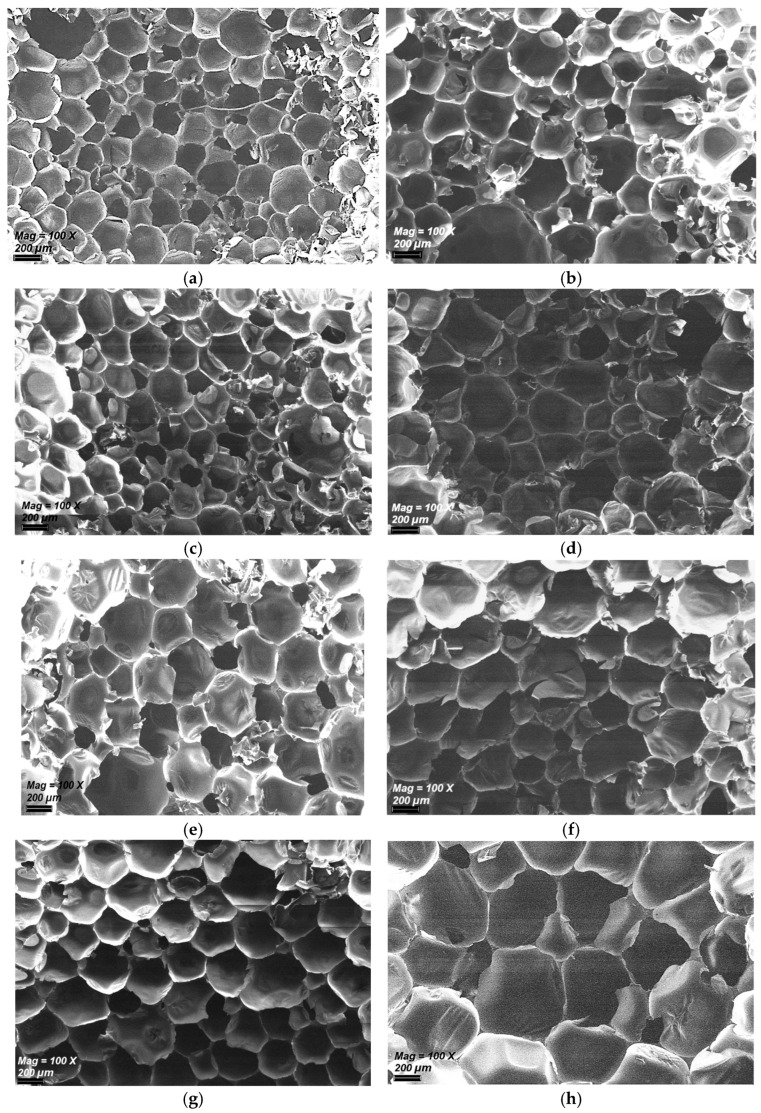
SEM of foam with 2% (**a**,**c**,**e**,**g**) and 3% (**b**,**d**,**f**,**h**) water content on 100 g of polyol B′ (**a**,**b**), C′ (**c**,**d**), Y′ (**e**,**f**) and Z′ (**g**,**h**).

Optical microscopy image analysis indicated that pores were spherical or oval (Table 6, Figure 3a–f). The larger diameter ranged from 278 to 424 µm, while the smaller one ranged from 252 to 403 µm. Wall thickness was in the range 14 to 33 µm. It has also been found that pores of PUFs obtained from compositions with 3% water were larger than those obtained with 2% water.

This is probably due to more CO_2_ being released during the former foaming process. The largest pores were obtained for PUF from Z′ polyol in the presence of 3% water per 100 g polyol, and consequently that PUF had the lowest apparent density among all obtained PUFs.

**Figure 4:**
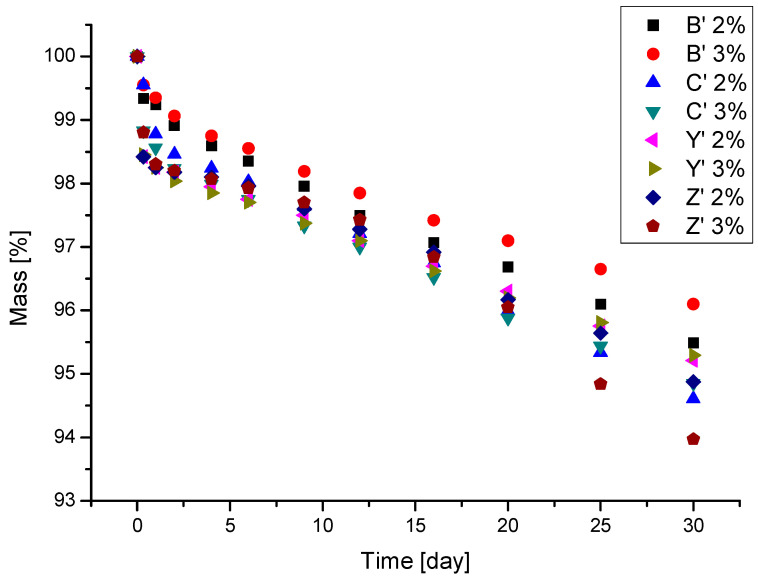
Mass loss of foams after heating at 150 °C obtained from polyols B′, C′, Y′ and Z′ (water content of 2% or 3% of the foaming compositions based on 100 g of polyol).

Thermal stability of PUFs was tested based on mass loss upon one month of thermal exposure at 150 and 175 °C, and on compressive strength before and after annealing (Table 7). All PUFs appeared to be thermally resistant at 150 °C. The mass was within the range of 3.9–6.0% upon one month of heating at 150 °C (Figure 4). The highest thermal stability was observed for PUF obtained from B′ polyol, namely 3.9–4.5%, depending on the amount of water used.

These values are comparable to, or even lower than, the weight losses observed for thermally resistant PUFs obtained from polyols containing azacyclic rings in their structure, such as perhydro-1,3,5-triazine or purine ring [31]. All obtained PUFs showed compressive strength values characteristic of rigid PUFs. The best compressive strength was shown by PUFs obtained from compositions with 2% water due to their higher apparent density and smaller pores. Annealing of PUFs at 150 °C improved their compressive strength, which we attribute to additional crosslinking taking place during annealing. Mass loss of PUFs originated mainly from physical processes, such as evaporation of trace amounts of TEA and previously absorbed water. Gradual degradation of PUFs occurred at 175 °C, which leads to 15.9–21.5% mass loss, comparable to that of PUFs containing an azacyclic component [31]. However, at this temperature, deformation of the obtained PUFs occurred due to their elastic character and the release of structural tensions. From this, we conclude that the obtained PUFs can be applied as insulating materials at temperatures up to 150 °C.

**Figure 5:**
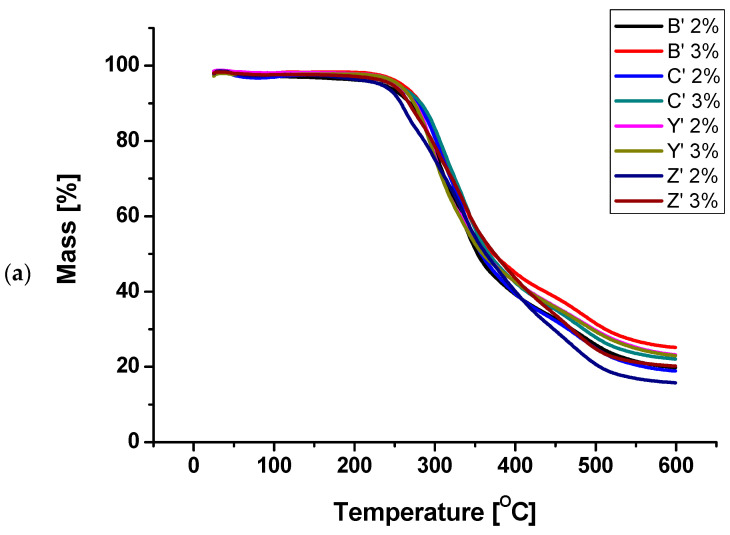

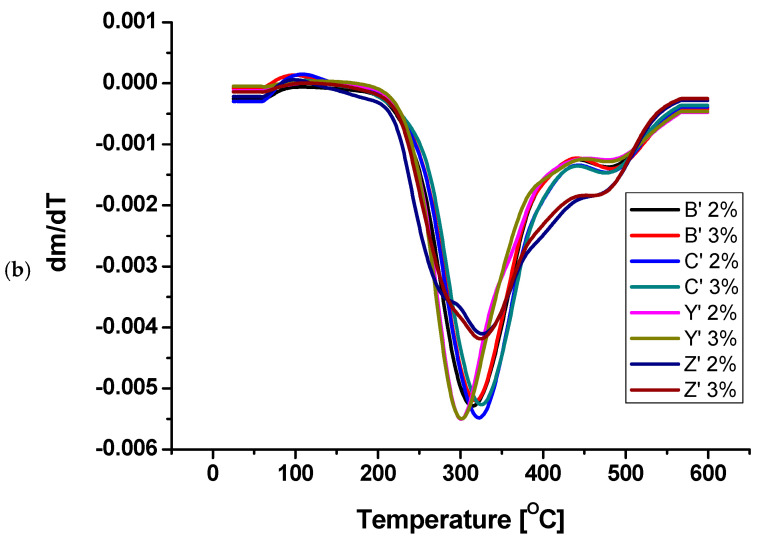
Thermal analysis of foams obtained from polyols B′, C′, Y′ and Z′: mass change as a function of temperature (**a**); differential mass change as a function of temperature (**b**). (Water content was 2% or 3% of the foaming compositions based on 100 g of polyol).

**Figure 6:**
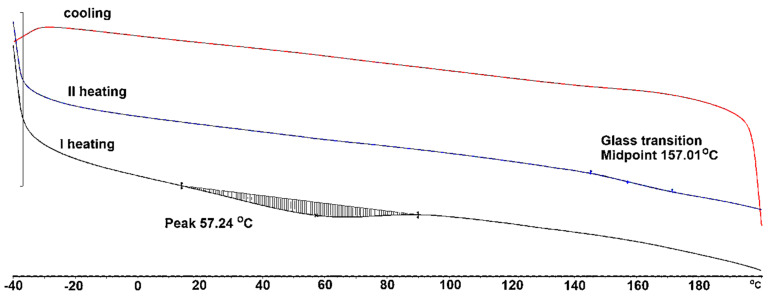
DSC thermogram of foam obtained from polyol Y’ with 2 wt% water.

Thermogravimetric analysis of obtained PUFs confirmed their high thermal resistance (Table 8, Figure 5a). The 5% mass loss temperature was within the range of 233–259 °C, while in one case it reached 346 °C. From the DTG curve (Figure 5b), we noticed an endothermic peak related to mass loss within 300–325 °C, which corresponds to polyurethane decomposition to amines and carbon dioxide [32]. The second, smaller peak was found at 480–500 °C, which corresponds to the dissociation of the methylene-aromatic ring bond introduced by pMDI [33,34]. Total thermal decomposition of the PUF was observed at ca 600 °C. According to DSC, all PUFs showed an endothermic peak in the range 10–90 °C during the first cycle, which was attributed to the evaporation of the catalyst and water. The effect was absent in the second cycle DSC (Figure 6). Therefore, the glass transition temperature was determined from the second cycle. The Tg was found within the range of 73–79 °C for PUFs obtained from polyols B′ and C′, whereas for the PUF obtained from polyol Y′ it was observed at 156–157 °C. These parameters indicate that the latter PUFs have not only high thermal resistance but also high heat resistance. The absence of Tg for some PUFs below 200 °C suggests that they remain in the glassy state throughout the entire analytical temperature region, which allows us to conclude that they are heat resistant.

### 3.3. Comparison of the Properties of PUFs Obtained from Different Oxyalkylated Polyols

In comparison with our previous studies [19,20], the present work introduces PC as a replacement for EC in the oxyalkylation of organosolv lignin. The properties of the obtained PUFs were compared with those of PUFs obtained by oxyalkylation of OL with a mixture of GL and EC (labeled B and C as described in [19]) and PUFs obtained by oxyalkylation of OL with a mixture of EC in GLYC (labeled Y and Z in [20]). Although the overall synthetic concept remains similar, the change of carbonate leads to noticeable differences in polyol structure and foam performance properties, particularly in terms of density, water absorption, and thermal stability.

The synthesis described in [19] is based on the reaction of a constant amount of OL with a mixture of GL and EC in various weight percentages, while the synthesis described in [20] used similar reactions and reagents except that GLYC was used as a component of the mixture. The results obtained here and previously are collected in Table 9. The values in brackets next to the symbols of polyols B and C as well as Y and Z show the weight ratio of structural fragments in the product: OL/GL/EC and OL/GLYC/EC (cf. Introduction). By comparison, the following conclusions can be drawn:

1. Polyurethane foams obtained from OL/GLYC/EC polyols are characterized by lower apparent density and water absorption than foams produced from OL/GL/EC polyols, while exhibiting a comparable thermal conductivity coefficient. Even lower apparent density and water absorption are shown by foams obtained with the participation of PC (both in the GL- and GLYC-based variants). Their thermal conductivity coefficient is even lower, reaching values typical of materials with good thermal insulation properties.
2. An advantage of foams obtained from OL/GLYC/EC polyols is that, despite their lower apparent density, they exhibit compressive strength comparable to that of foams obtained from OL/GL/EC polyols. This effect is even more pronounced in foams obtained with the participation of PC, which outperform, in this respect, foams based on polyols synthesized using EC.
3. The foams obtained in this work are characterized by significantly higher thermal resistance, assessed on the basis of mass loss, compared to conventional rigid polyurethane foams. At 150 °C, they exhibit slightly higher thermal resistance than foams based on OL/GLYC/EC polyols and significantly higher than foams obtained from OL/GL/EC polyols. This is evidenced by their two- or sometimes three-times lower mass loss after one month of exposure at this temperature.
4. Heating of foams obtained from OL/GL/EC, OL/GL/PC, and OL/GLYC/PC polyols at 150 °C always leads to an increase in their compressive strength. In contrast, foams obtained from OL/GLYC/EC polyols behave differently, as under these conditions they exhibit a decrease in compressive strength. The increase in strength in the first case results from better crosslinking of the foams due to the presence of GL in the synthesis. In the case of OL/GLYC/EC polyols, degradation predominates over crosslinking resulting in a decrease in compressive strength of the foams.
5. Based on the results obtained, we conclude that the PUFs obtained from polyols synthesized from lignin organosolv and PC in GLYC have the most practically useful properties at a temperature of up to 150 °C.

## 4. Conclusions

1. The method for the synthesis of organosolv lignin-derived polyols was developed through the reaction of lignin with propylene carbonate, glycidol, and glycerol. The products were obtained without the use of solvents and could be used directly in foaming compositions.
2. The obtained polyols were characterized by a hydroxyl number of approximately 400 mg KOH/g or higher, as well as physicochemical properties (viscosity, density, and surface tension) suitable for applications in rigid polyurethane foam technology. The use of propylene carbonate instead of ethylene carbonate made it possible to obtain polyols with more favorable processing properties and foams with improved performance characteristics.
3. It was found that under polyol synthesis conditions, some of the propylene carbonate decomposes into carbon dioxide and propylene oxide, which then isomerizes to a small extent into allyl alcohol.
4. The obtained foams exhibited an apparent density in the range of 39–56 kg/m^3^, a low thermal conductivity coefficient (0.0236–0.0270 W/m·K), and good dimensional stability. The lowest water absorption (0.53–0.60 vol.%) was observed for foams based on OL/GLYC/PC polyols.
5. Thermal analyses (TGA, DSC) confirmed the high thermal stability of the foams; they maintained operational stability up to 150 °C, while heat treatment at this temperature often led to an increase in compressive strength due to additional crosslinking of the structure.
6. Compared with previously reported systems based on ethylene carbonate [19,20], the use of propylene carbonate leads to the formation of polyols with lower viscosity and improved processing characteristics. Consequently, the resulting polyurethane foams exhibit lower density, reduced water absorption, and improved thermal resistance, confirming the advantage of PC-based systems over EC-based analogues.
7. The results of the study indicate that the most promising approach is the synthesis of polyols via the reaction of organosolv lignin with propylene carbonate in a glycerol medium, which enables the production of thermal insulation materials with high durability and potential applications in construction as well as in applications requiring enhanced thermal resistance.

## Figures and Tables

**Table 1:** Some properties of polyols at 20 °C.

Polyol	Reaction Time [h]	Temperature [°C]	Weight-Average Molecular Weight [u]	Density [g/cm^3^]	Viscocity [mPa s]	Surface Tension [mN/m]	Hydroxyl Number [mg KOH/g]	Acid Number [mg KOH/g]	Percentage of Lignin in the Product [%wt.]
B’	5	175–180	52,307	1.1528	6632	31.0	439	29.4	9.27
C’	5	180–185	76,370	1.1834	13,377	36.5	391	20.8	9.23
Y’	5	175	26,648	1.1200	769	32.8	564	37.0	11.38
Z’	4	180	21,247	1.1768	2439	36.6	497	27.0	11.28

**Table 2:** Interpretation of MALDI–ToF spectrum of polyol C′.

Entry	Signal Position *m*/*z*	Relative Intensity of Signal [%]	Molecular Ion Structure	Calc. Molecular Weight [g/mol]
1	81,156	1.5	AA + Na^+^	81,032
2	97,115	2.1	AA + K^+^	97,006
3	124,103	4.72	GLYC + MeOH	124,074
4	165,054	6.64	GL + OP + MeOH + H^+^	165,113
5	180,998	9.81	GLYC + OP + MeOH	182,115
6	197,063	7.16	AA + 2OP + Na^+^	197,115
7	229,158	1.76	2GL +OP +Na^+^	229,105
8	236,977	1.83	GLYC + GL + MeOH + K^+^	237,074
9	242,999	2.70	3GL − H_2_O + K^+^	243,063
10	252,940	4.12	AA + 3OP − H_2_O + K^+^	253,121
11	258,977	2.29	GLYC + 2OP + MeOH + H_2_O +H^+^	259,175
12	287,142	14.0	2GL + 2OP + Na^+^	287,147
13	289,157	12.83	AA + 4OP	290,209
14	303,123	14.28	AA + 3PO + MeOH + K^+^	303,157
15	345,168	11.22	2GL +3OP + Na^+^	345,189
16	347,159	14.58	GLYC + 3GL + MeOH + H^+^	347,192
17	359,136	10.22	3GL + 2OP − H_2_O + K^+^	359,147
18	361,143	100	AA + 4OP + MeOH + K^+^	361,199
19	363,163	67.10	GLYC + 4OP + K^+^	363,178
20	393,933	19.25	GLYC + 4OP + MeOH + K^+^	395,204
21	403,138	2.12	2GL + 4OP + Na^+^	403,230
22	409,101	7.21	4GL + OP + MeOH + Na^+^	409,204
23	417,132	11.96	3GL + 3OP − H_2_O + K^+^	417,189
24	419,188	68.82	3GL + 3OP + Na^+^	419,225
25	421,186	7.13	GLYC + 5OP + K^+^	421,220
26	435,106	8.58	GLYC + 5PO − H_2_O + MeOH + K^+^	435,235
27	437,206	10.54	GLYC +5OP + MeOK + Na^+^	437,272
28	477,175	12.57	3GL + 4OP + Na^+^	477,267
29	479,211	16.27	GLYC + 6OP + K^+^	479,262
30	493,175	25.13	2GL + 5OP + MeOH + Na^+^	493,298
31	495,236	18.02	GLYC + 6OP + MeOH + Na^+^	495,314
32	509,174	2.40	GLYC + GL + 5OP − H_2_O + MeOH + K^+^	509,272
33	551,231	6.48	2GL + 6OP + MeOH + Na^+^	551,340

Description of abbreviations used in the table: AA—allyl alcohol; GL—glycidol; GLYC—glycerol; OP—oxypropylene group; Na^+^—sodium cation; K^+^—potassium cation; −H_2_O—dehydration of the product; MeOH—methanol.

**Table 3:** Interpretation of MALDI–ToF spectrum of polyol Z’.

Entry	Signal Position *m*/*z*	Relative Intensity of Signal [%]	Molecular Ion Structure	Calc. Molecular Weight [g/mol]
1	81,158	6.8	AA + Na^+^	81,032
2	97,122	10.2	AA + K^+^	97,006
3	113,064	5.0	AA + MeOH + Na^+^	113,058
4	165,043	36.2	GLYC + MeOH + K^+^	163,037
5	180,995	27.0	GLYC +PO + MeOH	182,115
6	197,048	18.1	AA +2PO +Na^+^	197,115
7	231,157	25.1	AA + 3PO	232,167
8	245,126	6.6	AA + 2PO + MeOH + K^+^	245,116
9	247,138	39.6	GLYC + 2PO + K^+^	247,094
10	252,948	4.0	AA + 3PO − H_2_O + K^+^	253,121
11	258,988	3.8	GLYC +2OP + MeOH +H_2_O + H^+^	259,175
12	289,161	12.3	AA + 4PO	290,209
13	303,128	34.2	AA + 3PO + MeOH + K^+^	303,157
14	305,138	100	GLYC + 3PO + K^+^	305,137
15	321,125	33.9	AA + 4PO + MeOH	322,236
16	361,134	16.9	AA + 4PO + MeOH + K^+^	361,199
17	363,171	48.8	GLYC + 4PO + K^+^	363,178
18	377,143	19.4	GLYC + 4PO − H_2_O + MeOH + K^+^	377,194
19	395,159	13.1	GLYC + 4PO + MeOH + K^+^	395,204
20	421,162	2.7	GLYC + 5PO + K^+^	421,220
21	435,156	7.8	GLYC + 5PO − H_2_O + MeOH + K^+^	435,235
22	437,202	27.7	GLYC + 5PO + MeOH + Na^+^	437,272
23	453,180	22.3	GLYC + 5PO + MeOH + K^+^	453,246
24	469,190	2.4	AA + 7PO − H_2_O + Na^+^	469,314
25	502,797	2.3	AA + 7PO + K^+^	503,298
26	511,238	5.7	GLYC + 6PO + MeOH + K^+^	511,288
27	527,207	2.6	AA + 8PO − H_2_O + Na^+^	527,355

Description of abbreviations used in the table: AA—allyl alcohol; GLYC—glycerol; OP—oxypropylene group; Na^+^—sodium cation; K^+^—potassium cation; −H_2_O—dehydration of the product; MeOH—methanol.

**Table 4:** Optimal compositions and foaming parameters.

Polyol	Composition [g/100 g of Polyol]				Isocyanate Index	Foaming Process		
	Water	Silicon L-6900	TEA	pMDI		Cream Time [s]	Rise Time [s]	Tack Free Time [s]
B′	2	1.30	0.90	200	1.9	40	65	0
	3	1.00	0.35	223	2.1	40	120	0
C′	2	1.30	0.54	200	2.1	90	120	5
	3	1.30	0.40	227	2.3	55	145	4
Y′	2	1.55	0.63	245	1.8	45	110	0
	3	1.55	0.63	263	1.9	45	120	0
Z′	2	1.30	0.72	240	2.0	65	70	0
	3	1.30	0.72	260	2.1	90	100	5

**Table 5:** Some properties of foams.

Polyol	Amount of Water/100 g of Polyol	Density [kg/m^3^]	Absorption of Water [% vol.] After			Dimensional Stability [%] in Temperature 150 °C						Heat Conductance Coefficient [W/m∙K]
			5 min	3 h	24 h	Length Change After		Width Change After		Hight Change After		
						20	40 h	20 h	40 h	20 h	40 h	
B′	2	56.21 ± 1.32	0.24	0.33	2.24	1.08	1.34	1.08	1.46	0.64	0.97	0.0249 ± 0.0002
	3	47.65 ± 2.03	0.23	0.34	2.91	0.20	1.38	0.42	0.64	0.76	1.87	0.0264 ± 0.0001
C′	2	56.34 ± 1.60	0.20	0.92	4.93	2.00	2.00	1.01	1.97	0.40	0.47	0.0236 ± 0.0001
	3	54.05 ± 1.55	0.19	0.84	4.58	2.81	4.62	2.88	2.76	4.43	4.78	0.0248 ± 0.0001
Y′	2	53.60 ± 0.47	0.27	0.43	0.55	1.56	2.26	1.60	2.05	0.13	2.65	0.0267 ± 0.0001
	3	49.41 ± 1.41	0.31	0.46	0.54	1.76	1.94	1.21	1.38	0.95	1.56	0.0270 ± 0.0002
Z′	2	47.80 ± 1.46	0.42	0.45	0.53	0.28	0.56	0.64	0.88	0.92	1.25	0.0256 ± 0.0001
	3	39.11 ± 1.21	0.38	0.42	0.60	0.25	0.50	0.46	0.52	0.00	0.07	0.0255 ± 0.0001

The standard deviation in the case of absorption of water was 1.8%. The dimensional distortion did not exceed 2.4%.

**Table 6:** Results of micrograph analyses.

Foam Obtained from Polyol	Amount of Water/100 g of Polyol	Large Diameter [µm]	Smaller Diameter [µm]	Thickness of Cell Wall [µm]
B′	2	278 ± 70	254 ± 40	22 ± 2
	3	321 ± 45	288 ± 43	14 ± 2
C′	2	293 ± 40	252 ± 30	16 ± 3
	3	408 ± 39	352 ± 55	26 ± 5
Y′	2	424 ± 54	403 ± 44	33 ± 5
	3	383 ± 55	326 ± 53	32 ± 5
Z′	2	383 ± 50	339 ± 30	26 ± 3
	3	573 ± 82	522 ± 84	25 ± 3

**Table 7:** Mass loss and compressive strength of foams after one month exposure in high temperature.

Foam Obtained from Polyol	Amount of Water/100 g of Polyol	Mass Loss in %wt. After Exposure for a Month in Temperature		Compressive Strength [MPa]	
		150 °C	175 °C	Before Exposure	After Exposure in Temperature 150 °C
B′	2	4.51	17.65	0.267	0.357
	3	3.90	16.70	0.258	0.334
C′	2	5.39	21.50	0.346	0.336
	3	5.15	18.80	0.224	0.293
Y′	2	4.79	17.45	0.384	0.485
	3	4.71	15.87	0.225	0.418
Z′	2	5.12	19.52	0.342	0.442
	3	6.03	20.27	0.310	0.350

**Table 8:** Thermal analysis of foam determined by the dynamic method.

Foam Obtained from Polyol	Amount of Water/100 g of Polyol	T_5%_ [°C]	T_10%_ [°C]	T_25%_ [°C]	T_50%_ [°C]	Tg [°C]
B′	2	234	269	304	353	73.1
	3	259	283	313	373	-
C′	2	247	277	310	358	79.4
	3	250	282	316	367	-
Y′	2	255	275	300	362	156.5
	3	255	275	302	360	157.0
Z′	2	233	257	300	364	-
	3	346	268	309	374	-

**Table 9:** Comparison of some properties of foams obtained with the participation of OL.

Foam Obtained from Polyol	Water [%]	Density [kg/m^3^]	Absorption of Water [wt%] After 24 h	Heat Conductance Coefficient [W/m∙K]	Mass Loss in %wt. After Exposure for a Month in Temperature		Compressive Strength [MPa]	
					150 °C	175 °C	Before Exposure	After Exposure in 150 °C
B, OL/GL/EC = /1.00/3.75/12.50/	2	82.23	4.00	0.0353	23.11	42.41	0.263	0.420
	3	58.78	4.76	0.0355	21.43	41.45	0.201	0.214
C, OL/GL/EC = /1.00/5.00/10.00/	2	91.90	4.96	0.0339	20.75	39.68	0.211	0.254
	3	58.02	5.43	0.0366	20.14	38.97	0.221	0.254
Y, OL/GLYC/EC = /1.00/2.00/12.00/	2	57.68	0.52	0.0346	6.64	25.69	0.266	0.259
	3	48.14	0.57	0.0332	6.38	26.14	0.235	0.205
Z, OL/GLYC/EC = /1.00/4.00/8.00/	2	62.09	0.60	0.0409	6.08	21.14	0.533	0.425
	3	54.00	1.28	0.0366	6.27	22.90	0.367	0.362
B′, OL/GL/PC = /1.00/3.75/11.70/	2	56.21	2.24	0.0249	4.51	17.65	0.267	0.357
	3	47.65	2.91	0.0264	3.90	16.70	0.258	0.334
C′, OL/GL/PC = /1.00/5.00/8.70/	2	56.34	4.93	0.0236	5.39	21.50	0.346	0.336
	3	54.05	4.58	0.0248	5.15	18.80	0.224	0.293
Y′, OL/GLYC/EC = /1.00/2.00/5.91/	2	53.60	0.55	0.0267	4.79	17.45	0.384	0.485
	3	49.41	0.54	0.0270	4.71	15.87	0.225	0.418
Z′, OL/GLYC/EC = /1.00/4.00/3.18/	2	47.80	0.53	0.0256	5.12	19.52	0.342	0.442
	3	39.11	0.60	0.0255	6.03	20.27	0.310	0.350

## Data Availability

The original contributions presented in this study are included in the article. Further inquiries can be directed to the corresponding author.
